# 5-ALA Attenuates the Palmitic Acid-Induced ER Stress and Apoptosis in Bovine Mammary Epithelial Cells

**DOI:** 10.3390/molecules26041183

**Published:** 2021-02-23

**Authors:** Mst Mamuna Sharmin, Md Aminul Islam, Itsuki Yamamoto, Shin Taniguchi, Shinichi Yonekura

**Affiliations:** 1 Department of Biomedical Engineering, Graduate School of Medicine, Science and Technology, Shinshu University, Minamiminowa, Kamiina-gun, Nagano 399-4598, Japan; 18hb152b@shinshu-u.ac.jp (M.M.S.); 18hb151d@shinshu-u.ac.jp (M.A.I.); 2 Department of Biomedical Engineering, Graduate School of Science and Technology, Shinshu University, Minamiminowa, Kamiina, Nagano 399-4598, Japan; 19bs115g@shinshu-u.ac.jp; 3 Neopharma Japan Co., Ltd., Tokyo 102-0071, Japan; shint@neopharmajp.com; 4 Graduate School of Biosphere Science, Hiroshima University, Higashi-Hiroshima 739-8528, Japan; 5 Department of Biomolecular Innovation, Institute for Biomedical Sciences, Shinshu University, 8304 Minamiminowa, Kamiina, Nagano 399-4598, Japan

**Keywords:** 5-ALA, palmitic acid, bovine mammary epithelial cell, ER stress, oxidative stress

## Abstract

The conservation of mammary gland physiology by maintaining the maximum number of mammary epithelial cells (MECs) is of the utmost importance for the optimum amount of milk production. In a state of negative energy balance, palmitic acid (PA) reduces the number of bovine MECs. However, there is no effective strategy against PA-induced apoptosis of MECs. In the present study, 5-aminolevulinic acid (5-ALA) was established as a remedial agent against PA-induced apoptosis of MAC-T cells (an established line of bovine MECs). In PA-treated cells, the apoptosis-related genes *BCL2* and *BAX* were down- and upregulated, respectively. The elevated expression of major genes of the unfolded protein response (UPR), such as *CHOP*, a proapoptotic marker (C/EBP homologous protein), reduced the viability of PA-treated MAC-T cells. In contrast, 5-ALA pretreatment increased and decreased *BCL2* and *BAX* expression, respectively. Moreover, cleaved caspase-3 protein expression was significantly reduced in the 5-ALA-pretreated group in comparison with the PA group. The downregulation of major UPR-related genes, including *CHOP*, extended the viability of MAC-T cells pretreated with 5-ALA and also reduced the enhanced intensity of the PA-induced expression of phospho-protein kinase R-like ER kinase. Moreover, the enhanced expression of *HO-1* (antioxidant gene heme oxygenase) by 5-ALA reduced PA-induced oxidative stress (OxS). *HO-1* is not only protective against OxS but also effective against ER stress. Collectively, these findings offer new insights into the protective effects of 5-ALA against PA-induced apoptosis of bovine MECs.

## 1. Introduction

Dairy cows synthesize copious amounts of milk over the lactation period. Milk production is largely determined by the number of mammary epithelial cells (MECs) [1]. These cells are the structural and functional unit of the mammary gland and are indispensable for the synthesis and storage of milk before secretion from the udder. Hence, a reduction in the number of MECs via apoptosis can reduce the yield of milk. Therefore, maintaining the maximum number of active bovine MECs would ensure an optimum amount of milk production [2,3].

In the early lactation period, a negative energy balance (NEB) occurs in dairy cows when there is a discrepancy in the increase in nutrient requirements while the dry matter intake is decreased (up to 40%), due to fetal growth and hormonal change [4,5,6], which results in the mobilization of body fat and elevated circulating concentrations of nonesterified fatty acids to maintain energy requirements [7,8,9]. 

Palmitic acid (PA) is the most abundant saturated nonesterified fatty acid. Our previous study established that PA upregulated the expression level of the C/EBP homologous protein (CHOP) transcription factor via the protein kinase R-like ER kinase (PERK) arm of the unfolded protein response (UPR), which resulted in severe endoplasmic reticulum (ER) stress-induced apoptosis of bovine MECs [10]. PA has also been reported to induce oxidative stress (OxS) through the production of intracellular reactive oxygen species, which subsequently stimulate apoptosis of hepatocytes [11]. Therefore, PA might be an important factor in the reduction of MEC numbers as well as the milk yield in the early lactation period. Hence, a successful approach for inhibiting PA-induced apoptosis of MECs is needed as an efficient management tool to improve the lactation performance of dairy cows. 

5-Aminolevulinic acid (5-ALA), an endogenous amino acid of both animals and plants, is synthesized from glycine and succinyl coenzyme A via a condensation reaction in mitochondria with the help of ALA synthase [12]. A recent study by our group found that 5-ALA could reduce heat stress-mediated ER stress-induced apoptosis of bovine MECs [13]. 5-ALA also effectively inhibits apoptosis in response to OxS and other stressors [14,15,16,17]. Therefore, 5-ALA has the potential to protect bovine MECs, thereby facilitating an optimum amount of milk production. 

The objective of the current study was to examine the efficiency of 5-ALA in inhibiting ER stress-induced apoptosis of MECs in response to PA. Here, the viability of bovine mammary alveolar cells (MAC-T), a MEC line stably transfected with the large T-antigen of simian vacuolating virus 40, was assessed after pretreatment with 5-ALA followed by PA treatment. In addition, genes responsive to OxS and ER stress of PA-treated MAC-T cells with and without 5-ALA pretreatment were also identified.

## 2. Results

### 2.1. PA Reduced but 5-ALA Enhanced the MAC-T Cells Viability

**Figure 1:**
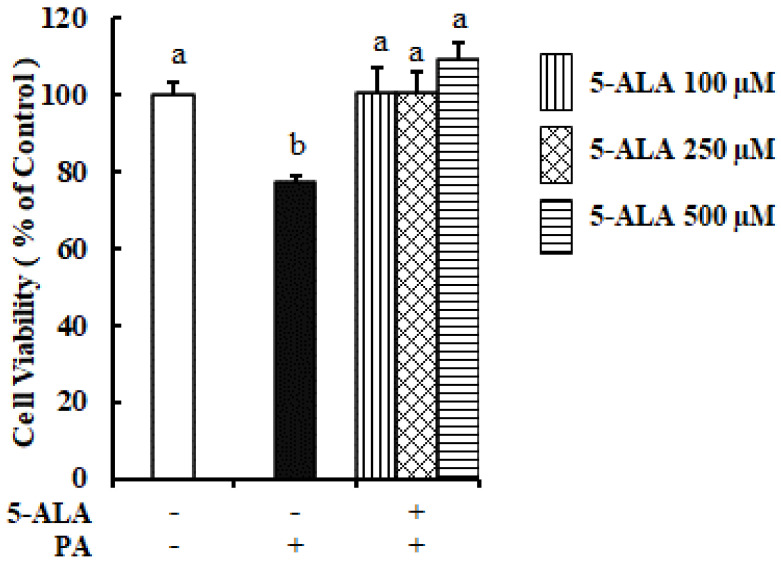
Measurement of the viability of bovine mammary alveolar cells (MAC-T) cells using 5-ALA pretreatment. 300 μM of PA with or without 5-ALA pretreatment at 100, 250 and 500 μM for 48 h were used to treat the confluent cells. The MTT assay was applied to determine the cell viability. Survival rates are expressed as percentages of the control cells. Three standard experiments were performed independently to show the data as the mean ± SEM (standard error of the mean), with different letters indicating significant differences at *p* < 0.05.

We previously reported that PA induced severe ER stress and reduced the viability of bovine MECs [7]. Consistent with the result of our previous study, PA also decreased the viability of MAC-T cells in the current study. The reduction of cell viability was 20% in the PA-treated group compared to the control one. Pretreatment with all doses of 5-ALA (100, 250, and 500 μM) significantly enhanced the viability of MAC-T cells, as compared with PA-treated cells (Figure 1). Therefore, 5-ALA has the potentiality to enhance the viability of MAC-T cells that was decreased by PA.

### 2.2. 5-ALA Reduced the Apoptosis Produced by PA in MAC-T Cells

**Figure 2:**
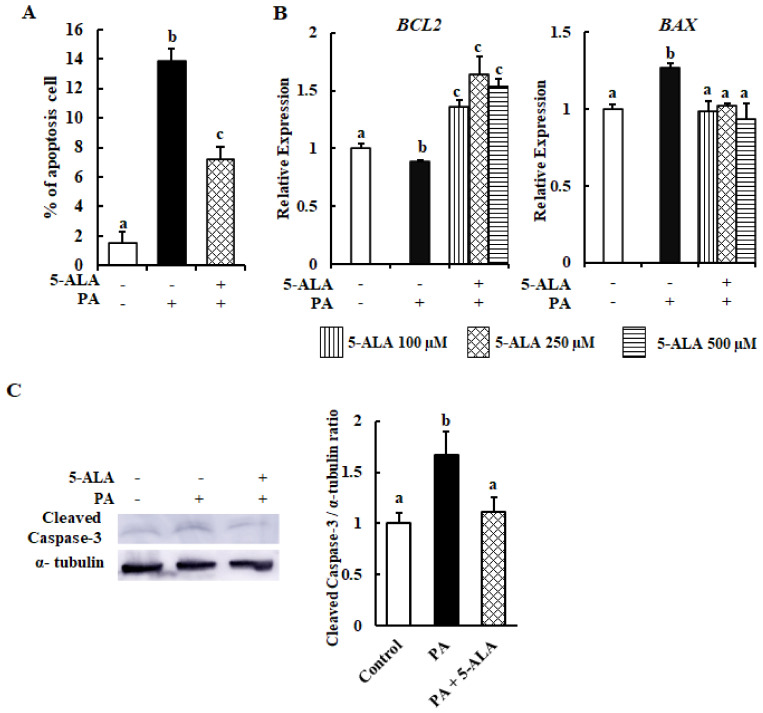
Assessment of the apoptosis rate and the expression levels of apoptosis-related genes from 5-ALA-pretreated MAC-T cells. (**A**) A FACS analysis was performed to assess the apoptotic rates of bovine MECs treated with 300 μM PA with or without 5-ALA pretreatment at 250 μM for 24 h. (**B**) Bovine MECs were treated with 300 μM PA with or without 5-ALA pretreatment at 100, 250 and 500 μM for 24 h. The BCL2 and BAX mRNA expression levels were determined by RT-qPCR and normalized to ACTB levels. Three standard experiments were performed independently to show the data as the mean ± SEM (standard error of the mean) with different letters indicating significant differences at *p* < 0.05. (**C**) MAC-T cells were served with 300 μM PA with or without 5-ALA pretreatment at 250 μM for 24 h, and the expression levels of cleaved caspase-3 and α-tubulin protein (internal control) were determined by a western blot analysis. Left: The data of three independent experiments of at least four replicates are shown. Right: Quantification of the cleaved caspase-3/α-tubulin ratio obtained by densitometric analysis. Three standard experiments were performed independently to show the data as the mean ± SEM (standard error of the mean), with different letters indicating significant differences at *p* < 0.05.

The rate of apoptosis was measured by flow cytometry analysis using the cells pretreated with 5-ALA and treated with PA. The apoptosis rate was 13.71% for cells treated with PA and 7.25% for those cotreated with PA and 5-ALA (Figure 2A). The results of the qRT-PCR analysis determined that *BCL2* and *BAX* were related to apoptosis. Expression of the antiapoptosis gene *BCL2* was downregulated, while that of the proapoptosis gene *BAX* was upregulated, respectively, in PA-treated cells, whereas *BCL2* expression was significantly upregulated and that of *BAX* was downregulated in cells pretreated with 5-ALA (Figure 2B). Moreover, cleaved caspase-3 protein expression was higher in the PA-treated group compared to the control, and it was significantly reduced in the 5-ALA-pretreated group in comparison with the PA group (Figure 2C). These data indicated that 5-ALA rescued MAC-T cells from PA-induced apoptosis.

### 2.3. 5-ALA Ameliorated the PA-Induced ER Stress

**Figure 3:**
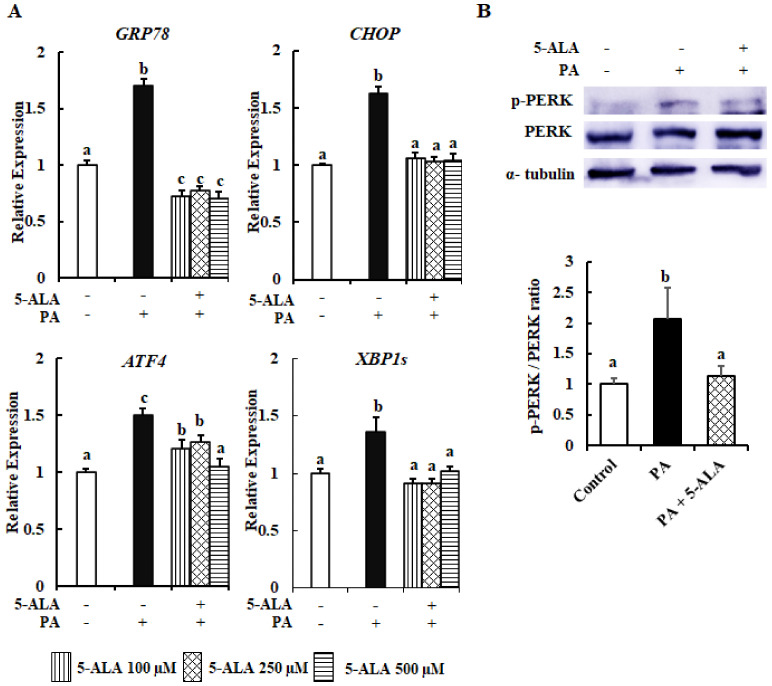
5-ALA reduced the expression levels of marker genes and a protein of PA-induced ER stress. Confluent MAC-T cells were treated with 300 μM PA with or without 5-ALA pretreatment at 100, 250 and 500 μM for 24 h. (**A**) The mRNA expression levels of *GRP78*, *CHOP*, *ATF4* and *XBP1s* were measured by RT-qPCR and normalized to *ACTB* levels. Three standard experiments were performed independently to show the data as the mean ± SEM (standard error of the mean). (**B**) Confluent MAC-T cells were stimulated with 300 μM PA with or without 5-ALA pretreatment at 250 μM for 24 h. The expression levels of phosphor-PERK, PERK and α-tubulin (internal control) were determined by western blot analysis. Upper: The data of three independent experiments of at least four replicates are shown. Lower: Quantification of the phospho-PERK/PERK ratio obtained by densitometric analysis. The data are presented as the mean ± SEM (standard error of the mean), with different letters indicating significant differences at *p* < 0.05.

The UPR-component genes *GRP78*, *CHOP*, *ATF4* and *XBP1s* expressions were examined as markers of ER stress in 5-ALA-pretreated MAC-T cells following treatment with PA. According to Figure 3A, PA treatment upregulated the expression levels of all of the above ER stress marker genes as compared with the control group, while pretreatment with 5-ALA downregulated the PA-induced upregulation of *GRP78*, *CHOP*, *ATF4* and *XBP1s*.

A western blot analysis of the phospho-PERK expression was performed to confirm the advantageous effect of 5-ALA. Figure 3B shows that the PA application increased the phospho-PERK expression but that it was reduced in cells pretreated with 5-ALA, indicating that 5-ALA could decrease PA-induced ER stress in MAC-T cells. Therefore, these data suggested that 5-ALA reduced PA-induced ER stress in MAC-T cells.

### 2.4. 5-ALA Culminated the PA-Induced OxS

**Figure 4:**
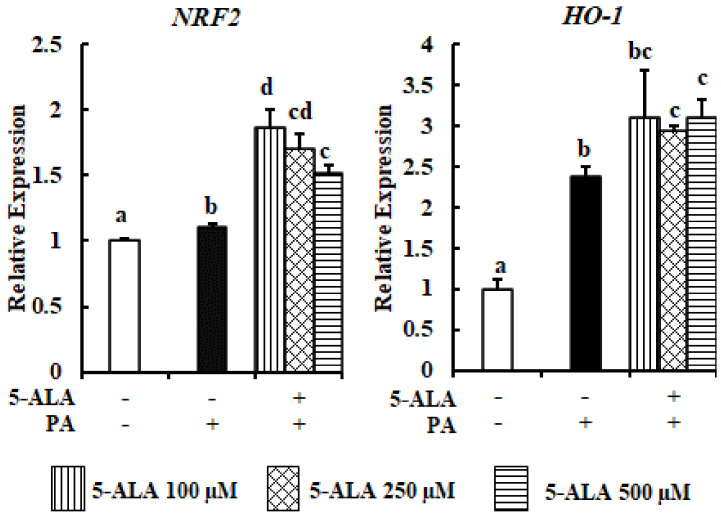
The effect of 5-ALA on the expression levels of OxS-related genes following PA treatment. Confluent MAC-T cells were treated with 300 μM PA with or without 5-ALA pretreatment at 100, 250 and 500 μM for 24 h. *NRF2* and *HO-1* mRNA expressions were quantified by RT-qPCR and normalized to *ACTB* levels. Three standard experiments were performed independently to show the data as the mean ± SEM (standard error of the mean), with different letters indicating significant differences at *p* < 0.05.

PA is a potent inducer of OxS in a variety of cell types [18,19]. The upregulation of NRF2, a master regulator of OxS and its target antioxidant gene HO-1 (heme oxygenase 1), by PA treatment was a marker of OxS. The stronger upregulation of NRF2 and HO-1 by 5-ALA pretreatment, as compared with PA treatment, was indicative of OxS (Figure 4). As a result, the enhanced expression of NRF2 and HO-1 in the 5-ALA-pretreated cell showed the protective antioxidant effect of 5-ALA for MAC-T cells.

## 3. Discussion

The data from our current study indicated that PA reduced viability by 20% and caused 13.71% apoptosis of MAC-T cells, while pretreatment with different doses of 5-ALA enhanced viability, consistent with the findings of previous studies [10]. We believe that the rest of the cell death is due to PA-induced necrosis. Unfortunately, we did not check whether PA mediated necrosis-related cell death. However, previous studies explained that PA induced necrosis and caused different types of cell death [20,21]. Therefore, we speculated that the remaining 6.29% of dead cells was due to necrosis in the PA-treated group. PA treatment decreased the expression of *BCL2* and increased that of *BAX*, indicating that PA augmented the proportion of apoptotic cells, while 5-ALA pretreatment had an opposite effect. Moreover, cleaved caspase-3 protein expression was significantly decreased by 5-ALA pretreatment. Overall, the proportion of apoptotic cells was decreased by cotreatment with 5-ALA and PA. Previous studies also reported that PA downregulated the expression of BCL2 and upregulated that of BAX and cleaved caspase-3, as markers of the induction of apoptosis [22,23]. However, 5-ALA blocked apoptosis and promoted the viability of MAC-T cells by increasing the expression levels of antiapoptotic genes.

In the present study, the mRNA expression of *GRP78* and *CHOP*, *ATF4* and *XBP1s*, and the phospho-PERK protein expressions were aggravated in PA-treated bovine MECs. GRP78, a major ER stress chaperon, acts to conserve ER homeostasis. Under physiological conditions, GRP78 binds to and inactivates all three UPR arms within the ER membrane. However, in response to ER stress, GRP78 disassociated from and activated all three sensors. Thereafter, phospho-PERK activated the transcription factors ATF4 and CHOP [24]. This CHOP expression has a negative correlation with the milk yield, as identified by an in vivo study [25]. Previous findings explained that PA treatment enhanced *GRP78* expression [26,27], which led to an imbalance in ER homeostasis. A recent study by our group found that PA induced severe ER stress-mediated apoptosis of MAC-T cells by increasing the *XBP1s*, *ATF4* and *CHOP* mRNA expression and the protein expression of phospho-PERK [10]. In contrast to PA treatment, pretreatment with 5-ALA decreased the expression levels of *GRP78*, *CHOP*, *ATF4* and *XBP1s*, as well as phospho-PERK. Therefore, 5-ALA has the potential to ameliorate PA-induced severe ER stress in MAC-T cells.

The findings of the current study also showed that *NRF2* and *HO-1* expressions by 5-ALA treatment were higher when compared with PA treatment only. Notably, the *NRF2* and *HO-1* expression levels were significantly upregulated in PA-treated cells as compared with control cells, which was compatible with a previous study [28]. Under nonstress conditions, in the cytoplasm, NRF2 binds with Kelch-like ECH-associated protein 1 (Keap1) to maintain NRF2 at a constant level of ubiquitination and degradation. Thus, a lower level of NRF2 is maintained [29]. In the case of OxS, due to the modification of the cysteine residue, Keap1 releases NRF2. The released NRF2 becomes activated and translocates into the nucleus. In the nucleus, NRF2 binds with an antioxidant response element and promotes the transcriptional activation of antioxidant genes, such as HO-1 [30]. Thus, the enhanced expression of *NRF2* and *HO-1* by PA treatment is a self-defense mechanism, yet insufficient to diminish PA-induced OxS. A previous study also demonstrated that the prolonged upregulation of *HO-1* with the use of an NRF2-specific activator compensated PA-induced OxS [28]. 

**Figure 5:**
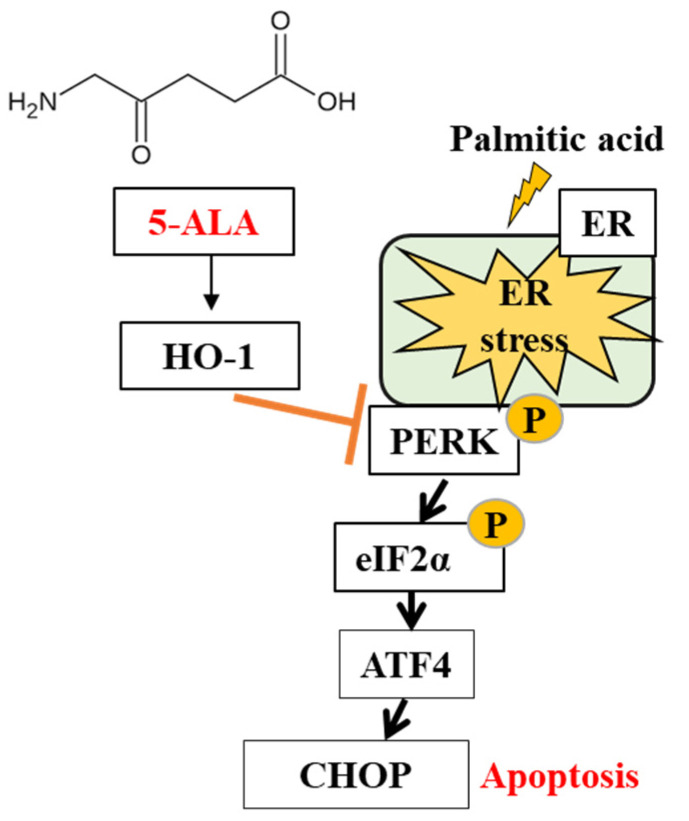
Schematic model of the suppression of palmitic acid-induced apoptosis in bovine MECs by 5-ALA.

A working model of the 5-ALA-mediated enhancement of *HO-1* in response to PA-induced ER stress is illustrated in the diagram presented in Figure 5. Here, the 5-ALA-instigated elevation of *HO-1* inhibited the PA-induced activation of phospho-PERK, which subsequently downregulated the expression of *ATF4* and *CHOP*. Therefore, the proportion of viable cells was increased, while that of apoptotic cells was decreased in the 5-ALA pretreatment group as compared with the PA treatment group. Accumulating evidence suggests that HO-1 upregulation is necessary for decreasing ER stress-induced apoptosis of hepatic [31], endothelial [32] and myocardial [33] cells. The enhanced expression of HO-1 suppressed the PA-mediated expression of phospho-eIF2α (downstream molecule of phospho-PERK) and CHOP to reduce apoptosis of HepG2 cells. Meanwhile, HO-1 silencing was unable to abrogate the upregulated expression of phospho-eIF2α and CHOP or to ameliorate apoptosis of hepatocytes [31]. Furthermore, several studies have reported the antiapoptotic effect of an increased HO-1 expression by the suppression of the PERK arm of the UPR [33,34,35]. Therefore, the increased mRNA expression of *NRF2* and *HO-1* by 5-ALA suggests that 5-ALA may act as an antioxidant response element to reduce OxS and ER stress and can effectively protect MAC-T cells from the detrimental effects of PA.

In summary, the results of the present study indicated that, as compared with PA, 5-ALA promoted the viability of bovine MECs by reducing the apoptosis rate via altering the expression levels of apoptosis-related genes. PA exerts severe ER stress and OxS in bovine MECs. In contrast, 5-ALA reduces the expression levels of UPR component genes and proteins to circumvent PA-mediated ER stress. Moreover, prolonged *HO-1* expression by 5-ALA was not only operative against OxS but also suppressed the signaling of the PERK pathway of the UPR. Thus, 5-ALA pretreatment is beneficial to bovine MECs as well as to udder physiology.

## 4. Materials and Methods

### 4.1. Reagents

Neopharma Japan Co. Ltd. (Tokyo, Japan) has provided the 5-ALA. Fetal bovine serum (FBS) was collected from Equitech-Bio (Cotton Gin Lane, TX, USA). Dulbecco’s Modified Eagle Medium (DMEM) was bought from Invitrogen (Carlsbad, CA, USA). Penicillin, streptomycin, hydrocortisone and bovine insulin were bought from Sigma-Aldrich (St. Louis, MO, USA). All other compounds were purchased from Nacalai Tesque (Kyoto, Japan).

### 4.2. Cell Culture and Treatment

Immortalized bovine MECs (MAC-T cells) were generously gifted by Dr. Sangun Roh of Tohoku University, Sendai, Japan. Cells were cultured in DMEM containing 10% FBS, 1% penicillin and streptomycin, 5 μg/mL bovine insulin, and 1 μg/mL hydrocortisone. The cell culture temperature was 37 °C under 5% CO2. 5-ALA was added to MAC-T cells for 48 h before PA treatment to examine its activity in preventing palmitic acid (PA)-related damage. PA solution was prepared as previously described by Sharmin et al. [10]. The preserved PA stock solution in 0.1% BSA was added to the cell culture medium before treatment to obtain the desired final concentration of 300 μM. 

### 4.3. Cell Viability Test

The viability of MAC-T cells was measured using an MTT Cell Viability Assay Kit (Biotium, Fremont, CA, USA), following the instructions of the manufacturer. Briefly, MAC-T cells were seeded in 96-well plates at a density of 1 × 10^3^ cells per well. MAC-T cells were pretreated with 5-ALA (100, 250 and 500 μM) followed by PA challenged for 48 h. Then, MTT solution was added at a rate of 10 μL/100 μL of culture medium and incubated for 4 h at 37 °C. Last, after the addition of 200 μL of dimethylsulfoxide (DMSO)/well, an absorbance reading was taken at 570 nm with a reference wavelength of 630 nm using a multimode microplate reader (iMark microplate reader, Bio-Rad, Hercules, CA, USA).

### 4.4. RNA Extraction and Quantitative Real-Time PCR

The entire RNA was extricated from MAC-T cells by applying TRIzol (Invitrogen), maintaining the protocol given by the manufacturing company. cDNA was synthesized from gross RNA by utilizing gDNA Remover, requiring qPCR RT Master Mix (Toyobo, Osaka, Japan). SYBR Premix Ex Taq^TM^ II (TaKaRa Biotechnology, Kusatsu, Japan) was applied for the quantitative real-time PCR analysis. Primer sequences for the quantitative PCR were used according to Islam et al. [13] and Sharmin et al. [10]. The relative mRNA expression was calculated by the 2^−∆∆CT^ comparative method using β-actin (*ACTB*) expressions and was expressed as values relative to control. The amplification of serial cDNA dilutions was performed to examine the sensitivity of reactions and the magnification of contaminating products, such as extensions of self-annealed primers. The manufacturer’s instructions were maintained to perform the data analysis.

### 4.5. Western Blot Analysis

After washing, radioimmunoprecipitation assay (RIPA) lysis buffer was added to the cells. The composition of RIPA lysis buffer was 50 mM Tris-HCl, 1 mM EDTA, 150 mM NaCl pH 7.4, 0.05% SDS, 0.2% sodium deoxycholate and 1% Tergitol-type NP-40 with a protease inhibitor cocktail (Nacalai Tesque). Following centrifugation (10 min at 20,000× *g*), the protein concentrations were evaluated using a Bio-Rad Protein Assay Kit (Bio-Rad Laboratories, Hercules, CA, USA). SDS-PAGE was executed using 60 μg of cellular extracts on 4–20% polyacrylamide gels, and polyvinylidene difluoride (PVDF) membranes were used to transfer (semidry transfer) the protein from gel to membrane. Membranes were then incubated for 1 h in 1% PBT and 0.01% Tween 20 solution containing 4% skim milk powder (blocking buffer). Afterwards, membranes were incubated at room temperature with antiphosphorylated PERK (Santa Cruz Biotechnology (sc-32577), Santa Cruz, CA, USA), anti-PERK (Santa Cruz Biotechnology (sc-13073)), anticleaved caspase-3 (Cell Signaling Technology (9661), Danvers, MA, USA) and anti-α-tubulin (MBL Co., Nagoya, Japan) antibodies diluted in blocking buffer. Next, antirabbit IgG secondary antibody (GE Healthcare, Pittsburgh, PA, USA) was used for the incubation of the membranes. The final working ratio of the antibody dilution was 1:500 for antiphosphorylated PERK and anti-PERK; and 1:1000 for anticleaved caspase-3 and anti-α-tubulin. An ECL Prime Western Blotting Detection Reagent Kit (GE Healthcare) was utilized for the visualization of the enhanced chemiluminescent (ECL) membranes, and images were captured using an Image Quant LAS 500 (GE Healthcare). The images were analyzed with the ImageJ software from the NIH.

### 4.6. Apoptosis Rate Analysis by Flow Cytometer

The apoptosis rate was determined using a tunnel assay with an in situ apoptosis detection kit (TaKaRa Bio Inc., Kusatsu, Shiga, Japan) according to the manufacturer’s instruction. Briefly, cells were incubated with 4% paraformaldehyde at room temperature for 15–30 min for fixing up. Afterwards, buffer permeabilization cells were stained with TdT (Terminal deoxynucleotidyl Transferase) for 60–90 min at 37 °C. Finally, TdT positive cells were analyzed using the SH800S cell sorter (Sony Biotechnology Inc., San Jose, CA, USA).

### 4.7. Statistical Analysis

Student’s *t*-test was utilized to measure the statistical significance to make a comparison between two samples. For multiple comparisons, an ANOVA with a post hoc Tukey–Kramer’s honestly significant difference test was performed. Statistically significant was considered to be *p* < 0.05.

## Data Availability

The data presented in this study is available within the article.

## Sample Availability

Samples of the compounds are not available from the authors.
